# The Health of Nations Fund: Financing global drug development

**DOI:** 10.1371/journal.pgph.0005248

**Published:** 2026-06-16

**Authors:** Joonhyuk Cho, Manish Singh, Chaoyi Zhao, Shomesh E. Chaudhuri, Andrew W. Lo

**Affiliations:** 1 Laboratory for Financial Engineering, Massachusetts Institute of Technology, Cambridge, Massachusetts, United States of America; 2 Computer Science and Artificial Intelligence Laboratory, Massachusetts Institute of Technology, Cambridge, Massachusetts, United States of America; 3 Department of Electrical Engineering and Computer Science, Massachusetts Institute of Technology, Cambridge, Massachusetts, United States of America; 4 Sloan School of Management, Massachusetts Institute of Technology, Cambridge, Massachusetts, United States of America; 5 QLS Technologies LLC, Cambridge, Massachusetts, United States of America; 6 Santa Fe Institute, Santa Fe, New Mexico, United States of America; London School of Economics and Political Science, UNITED KINGDOM OF GREAT BRITAIN AND NORTHERN IRELAND

## Abstract

We explore the design and performance of a “Health of Nations Fund,” a proposed securitization of a biomedical “megafund” that pools capital to invest in a diversified set of global drug development projects. Incorporating assets from four stages of the drug development process as well as royalties from approved therapeutics across eight therapeutic areas, our Monte Carlo simulations show that such a megafund can deliver an annual expected return of 12.0% with a Sharpe ratio of 1.37, indicating a favorable balance between risk and return. At the same time, it finances an average of 25 approved drugs that potentially benefit approximately 44 million patients worldwide over a 14.5-year horizon. To enhance the fund’s financial and therapeutic value, we integrate an optimization framework into our design that accounts for global disease prevalence and severity, allowing investors to adjust the allocation of capital according to their preferences for financial performance and healthcare impact. Sensitivity analyses indicate that this megafund typically generates double-digit annual returns across most scenarios.

## 1. Introduction

Investing in biomedical innovation involves navigating a sea of uncertainty—the required investments are significant, gestation lags are long, and payoffs are highly uncertain. The risks include not only scientific risks but also regulatory risks of drug approval and financing risks [1,2]. Traditional financing models have generally struggled to support early-stage drug development, which corresponds to the riskiest and most challenging part of the drug approval process. Due to the lack of funding, early-phase translational research is often referred to as the “Valley of Death” in the drug development pipeline, failing to attract investors and leading to a bottleneck in biopharma R&D productivity in recent years [3–5].

As a consequence, the idea of forming diversified drug development portfolios is gaining more traction [6,7]. A portfolio represents a group of drug development projects whose risks and potential returns are assessed collectively rather than individually. The rationale is straightforward: by spreading investments across many development programs, investors can reduce risk while capturing the financial upside of scientific breakthroughs across a broad range of therapeutic areas. Successful breakthroughs in these areas would yield significant improvements in quality of life and reduced mortality worldwide as well as returns for investors.

In this article, we build on these ideas by proposing a “Health of Nations Fund” (HNF), a securitization of a globally diversified portfolio of biomedical projects. The intention is to pool capital on a global scale to invest in a diversified portfolio of drug development programs, which are also sourced globally. Fernandez, Stein, and Lo [8] originally proposed a megafund portfolio approach for raising investment in the drug development process. In their framework, the megafund is financed using two standard forms of capital: equity and debt. Equity represents an ownership stake in the megafund, with equity investors sharing in both the profits from successful projects and the losses from unsuccessful ones. Debt is raised through the issuance of research-backed obligations (RBOs), debt securities whose coupons and principal are backed by the future cash flows generated by the megafund’s portfolio of drug development projects. RBO investors would typically face lower risk and receive more predictable income streams than equity investors. Using this financing structure, Fernandez, Stein, and Lo [8] demonstrate the theoretical feasibility of the portfolio-based approach and report simulated average investment returns of 8.9–11.4% for equity holders and 5–8% for RBO holders, based on a megafund size of $5–15 billion.

More recently, Lo and Siah [9] modified the megafund framework of Fernandez, Stein, and Lo [8] to make it more realistic by relaxing some of the assumptions (such as the independence of clinical trial projects) and using more accurate parameters (including cost, duration, and the probability of success (PoS) of clinical trials). This megafund structure has also been proposed as a funding model for several targeted therapeutic areas, including rare and orphan diseases [10], Alzheimer’s disease [11], pediatric oncology [12], ovarian cancer [13], brain cancer [14], and opioid use disorder [15]. However, most of these studies focus on a single therapeutic area and construct portfolios of drug development assets in the preclinical phase.

Beyond these theoretical models, a real-world example demonstrating its practical feasibility for global health is the Global Health Investment Fund (GHIF). GHIF is a $108 million social impact fund launched in 2013 with blended public, philanthropic, and private capital to finance the late-stage development of drugs, vaccines, diagnostics, and devices targeting diseases that disproportionately burden low- and middle-income countries. It manages a tight portfolio of roughly a dozen companies, using tools such as partial-loss protection to balance profit with social purpose [16]. While GHIF demonstrates that a diversified fund for global health is feasible in practice and has supported more than a dozen products reaching hundreds of millions of people [17], its scale and late-stage focus differ from the larger, multi-stage, multi-therapeutic securitization structure that we propose for the HNF.

This article extends the framework of Lo and Siah [9] to further embody the principle of “not putting all your eggs in one basket” by including assets from various stages of the drug development process and across several therapeutic areas. In particular, we include assets from four stages of drug development (preclinical, phase 1, phase 2, and phase 3) and royalties from approved therapeutics. The riskiest stage—the preclinical phase—has the lowest PoS (around 10–15%) but offers the potential for substantial returns upon successful approval. In contrast, royalty investments provide relatively stable and less risky cash flows, yielding correspondingly lower returns. We propose diversifying the portfolio across eight therapeutic areas: oncology, metabolic/endocrinology, cardiovascular, central nervous system, autoimmune/inflammation, genitourinary, infectious disease, and ophthalmology. These therapeutic areas include the majority of the leading global causes of disability and death.

From a healthcare perspective, our HNF is also designed to ease financing constraints that often prevent clinically promising programs from advancing. By pooling assets across multiple therapeutic areas and stages of development, the structure combines relatively stable cash flows—such as those from later-stage or royalty investments—with high-variance early-stage projects. This balance not only increases the likelihood of successful regulatory approvals but also reduces vulnerability to setbacks in any single domain. In turn, this is expected to lead to more completed clinical trials, more therapies approved, and earlier global patient access across a wide range of diseases. Thus, the megafund framework will align investor returns with global public health benefits—a model of “doing well by doing good.”

Our global focus further extends the megafund framework of Fernandez, Stein, and Lo [8] in several respects. First, the broader set of therapeutic areas provides greater diversification, yielding more attractive risk-adjusted returns for investors and, consequently, larger amounts of capital drawn to the HNF. Second, since the publication of Fernandez, Stein, and Lo’s paper [8], the biopharma sectors in several countries, such as China and India, have grown in size and sophistication, creating new sources of potentially attractive investment opportunities for the HNF. Third, international scientific and medical collaboration was dramatically accelerated by the COVID-19 global pandemic, creating a valuable network of expertise that the HNF can leverage. And finally, because many countries have both a single-payer healthcare system and a sovereign wealth fund, there are opportunities for a globally focused investment fund to raise substantial capital from a country and offer more than just a return on capital—it can provide access to life-saving drugs in exchange for advance purchase agreements that lock in attractive pricing in exchange for reducing a drug’s launch risk. This feature would have been particularly useful for many countries during the COVID-19 pandemic.

Our design process for the HNF begins with an equal-weighted megafund portfolio of $12.5 billion. Although substantial, this amount is modest in the context of global pharmaceutical R&D: recent industry and policy reports estimate worldwide spending to be on the order of hundreds of billions of U.S. dollars per year [18]. A $12.5 billion HNF would therefore represent only a small share of global pharmaceutical R&D investment, even though it would be large relative to existing global-health product development vehicles and several times greater than the roughly $4 billion invested annually in neglected-disease R&D [19].

Using Monte Carlo simulation, we find that, with a debt-to-equity ratio of 7:3, the megafund can achieve an expected annualized equity return of 12.0% and an annualized Sharpe ratio of 1.37 for investors, where the Sharpe ratio is a standard measure of risk-adjusted return that summarizes how much excess return an investment delivers per unit of volatility. In other words, the portfolio is expected to earn 1.37 units of excess return for each unit of return volatility. On average, the megafund also leads to the approval of 24.7 drugs across therapeutic areas that would potentially benefit approximately 44.0 million patients worldwide over 14.5 years. These findings demonstrate both the feasibility of the diversified megafund approach for drug development and its viability as an attractive investment vehicle.

We then extend our equal-weighted megafund portfolio by allowing investors to choose portfolio weights across different development stages and therapeutic areas. In particular, we use the disability-adjusted life year (DALY)—a measure of overall disease burden, expressed as the cumulative number of years lost due to ill health, disability, or early death—as a measure of the global prevalence and severity of disease, and propose an optimization framework that explicitly incorporates DALYs into its objective function, balancing financial performance, risk mitigation, and healthcare impact. Through this joint optimization, we show that the optimized megafund outperforms the equal-weighted portfolio across both financial returns and healthcare impact. Moreover, the framework is flexible, allowing investors to adjust the trade-off between financial performance and healthcare outcomes according to their preferences.

Finally, to explore how individual parameters affect the overall performance of the fund, we conduct a sensitivity analysis by varying key simulation inputs, including the debt-to-equity ratio, the PoS in drug development, the expected market value of drugs, and the cost of capital of the projects. This analysis allows us to assess how changes in these factors affect the fund’s viability and its returns. Our results show that, in most cases—except when drug market values are reduced by 50% and the cost of capital is as low as it was in 2021—the fund consistently delivers double-digit annual returns, demonstrating the robustness and attractiveness of the megafund approach across different scenarios and assumptions.

Of course, real-world drug development is influenced by factors that go beyond our stylized model, including multi-country regulatory requirements, site activation and patient recruitment constraints, and pricing and reimbursement uncertainties. In Section 4, we examine how such operational and regulatory realities may affect the performance and management of the HNF and how our megafund framework should be interpreted in light of these complexities.

Compared to prior megafund studies, our portfolio selection framework differs in two important ways. First, rather than acquiring only preclinical assets that must progress through successive trial phases before approval, we diversify the megafund across multiple stages of drug development. This approach balances risk and return, stabilizes interim cash flows, and allows the fund to better manage upfront debt obligations, thereby enhancing both resilience and long-term profitability. Second, we incorporate royalty assets, which were not considered in earlier studies. Royalties provide relatively predictable cash flows that help meet debt obligations consistently, enabling greater leverage and higher expected returns for equity investors in turn. Together, these innovations strengthen the financial sustainability of the megafund while maintaining its potential to support drug development on a truly global scale.

In Section 2 we introduce the HNF framework. We present the financial performance and healthcare impact of our megafund, along with sensitivity analyses, in Section 3. Section 4 discusses the limitations and practical considerations of our study. We conclude in Section 5, and provide a detailed description of the parameter setup used in our simulations in S1 Text.

## 2. Methods

The HNF is a megafund consisting of a large number of drug development projects with varying risk and reward characteristics. It serves both as a security for investors, backed by drug assets, and as a funding vehicle for global drug development projects. In Sections 2.1, 2.2, and 2.3, we introduce the structure, the portfolio construction, and the simulation framework of our HNF, respectively.

### 2.1 Megafund structure

Table 1 shows the capital structure and asset allocation methodology of the HNF. The design combines debt and equity financing with a diversified portfolio of drug development projects and royalty assets.

**Table 1:** Capital structure and asset allocation of the Health of Nations Fund.

Capital Structure				
Component	Total	Senior Debt	Junior Debt	Equity
Amount ($ million)	12,500	7,500	1,250	3,750
Tenor (Years)	14.5			
**Asset Allocation**				
Category	Total ($ million)	Details		
Drug Development	10,100	4 stages × 8 therapeutic areas = 32 categories		
Royalty Assets	2,400	12 assets		

#### Target fund size.

The fund size is set at $12.5 billion, which we take as our target amount. In practice, however, it is difficult to invest in arbitrary fractions of drug development projects. As a result, the actual fund size may differ slightly from the target. For example, if the target allocation is $10 million and each project costs $3 million, the fund can only acquire three projects, for a total of $9 million. We further discuss this in Section 2.3.

#### Capital structure.

Consistent with prior megafund studies, we assume a leveraged structure consisting of senior debt, junior debt, and equity in a ratio of 60:10:30. Coupon rates are set at 5% for senior debt and 10% for junior debt, reflecting their relative risk profiles as well as the high interest rate environment. Equity serves as the residual tranche and absorbs the upside potential of the fund after satisfying all debt obligations. The overall tenor of the fund is 14.5 years, which aligns with the expected time horizon for clinical trial progression and asset liquidation.

#### Asset allocation.

The HNF invests the total $12.5 billion over the 14.5-year horizon across two major asset categories: drug development projects and royalty assets. Of this, $2.4 billion is allocated to 12 royalty assets, which provide relatively stable cash flows that help the fund meet ongoing debt obligations. Each royalty asset is acquired at a cost of $200 million and generates cash flows over a 10-year period, since drug patents typically last for about 10 years. A detailed description of the cash flows from royalty assets used in our simulation is provided in Table H in S1 Text. The remaining $10.1 billion is distributed across drug development programs spanning four clinical stages (preclinical, phase 1, phase 2, and phase 3) and eight therapeutic areas (oncology, metabolic/endocrinology, cardiovascular, central nervous system, autoimmune/inflammation, genitourinary, infectious disease, and ophthalmology). This results in 32 distinct stage–area investment categories.

To evaluate the performance of the megafund, we construct two types of portfolios. The first is an equal-weighted portfolio across all stage–area categories, which serves as a benchmark. The second is an optimized portfolio that explicitly incorporates healthcare impact in addition to financial performance. The optimization methodology is described in Section 2.2.

### 2.2 Optimal portfolio construction

We determine the portfolio weights for the 32 distinct stage–area categories by jointly considering three dimensions: expected return, risk, and healthcare impact. First, the expected return reflects the projected financial performance of the portfolio. Second, diversification across therapeutic areas and clinical phases mitigates outcome risk. Third, healthcare impact measures the potential global improvement in patient health generated by the portfolio. Balancing these three dimensions allows us to optimize capital allocation not only for financial efficiency but also for maximizing global social benefit.

We formalize this optimization problem as follows. Let i=1,2,…,32 represent different stage–area categories, each comprising multiple drug development projects. On average, drug development projects in category *i* require an acquisition cost of *V*_*i*_ and yield a market value of *X*_*i*_ after *T*_*i*_ years if the drug is approved, and nothing otherwise.

The status of the *k*-th project in category *i* can be denoted by a Bernoulli random variable, *I*_*i*,*k*_ ∼ Bernoulli(*p*_*i*_), where *p*_*i*_ represents the average probability of approval for projects in category *i*. When *I*_*i*,*k*_ = 1, the drug is approved; when *I*_*i*,*k*_ = 0, the project fails. Therefore, the payoff of this project is given by *X*_*i*_*I*_*i*,*k*_. Project outcomes are uncertain at year 0 and are only revealed at year *T*_*i*_.

Assume that investors in the megafund use a hurdle rate of *r*_*h*_ to evaluate investment performance. Under this framework, the profitability index (PI) of the *k*-th project in category *i*—defined as the present value of future revenues, $X_{i}I_{i,k}/(1+r_{h}{)}^{T_{i}}$, divided by the initial investment cost, *V*_*i*_—is given by


$$\begin{array}{c} {PI}_{i,k}=\frac{X_{i}I_{i,k}}{V_{i}(1+r_{h}{)}^{T_{i}}}. \end{array}$$

Therefore, the expected PI and its variance are


$$\begin{array}{c} \mu_{i}=\frac{X_{i}p_{i}}{V_{i}(1+r_{h}{)}^{T_{i}}},\;\sigma_{i}^{2}=\frac{X_{i}^{2}p_{i}(1-p_{i})}{V_{i}^{2}(1+r_{h}{)}^{2T_{i}}}. \end{array}$$ (1)

In our analysis, we adopt a hurdle rate of 9.045%, which corresponds to the average cost of capital in the drug development industry as estimated by Aswath Damodaran using data as of January 2025 [20].

We construct a portfolio with weights $w=(w_{1},w_{2},\ldots ,w_{32}{)}^{⊤}$, where *w*_*i*_ denotes the weight assigned to category *i*. When constructing the optimal portfolio, we take into account three key factors: expected return, variance, and healthcare impact.

#### Expected return.

The expected PI of the portfolio is given by


$$\begin{array}{c} \text{Expected PI}=w^{⊤}\mu =\sum_{i=1}^{32}w_{i}\mu_{i}, \end{array}$$ (2)

where $\mu =(\mu_{1},\mu_{2},\ldots ,\mu_{32}{)}^{⊤}$ is the vector of expected PI for all categories, as defined in Eq (1).

#### Variance.

The variance of the portfolio is given by


$$\begin{array}{c} \text{Variance}=w^{⊤}\Sigma w, \end{array}$$ (3)

where Σ is the covariance matrix between different stage–area categories. The variance of each category is given in Eq (1), and the correlations between different categories are set according to Section G in S1 Text.

#### Healthcare impact.

The healthcare impact of the portfolio is given by


$$\begin{array}{c} \text{Healthcare impact}=w^{⊤}h, \end{array}$$ (4)

where $h=(h_{1},h_{2},\ldots ,h_{32}{)}^{⊤}$ is the vector of healthcare impact values for all categories. The healthcare impact of stage–area category *i*, denoted *h*_*i*_, is defined as


$$\begin{array}{c} h_{i}:=\frac{{\text{DALY}}_{i}}{V_{i}/p_{i}}, \end{array}$$

where DALY_*i*_ is the average DALY of the therapeutic area corresponding to category *i* obtained from the 2021 Global Burden of Disease study [21]. The denominator, *V*_*i*_/*p*_*i*_, represents the expected cost of a successful trial in category *i*. Therefore, *h*_*i*_ can be interpreted as the total DALYs of all patients potentially affected by projects in category *i* per dollar invested. A larger *h*_*i*_ implies a stronger healthcare impact for trials in that category. The values of DALYs used in this study are given in Table I in S1 Text.

We use DALYs in this construction because they provide a standardized and widely used measure of disease burden that combines years of life lost and years lived with disability into a single summary metric, allowing us to compare potential health gains across therapeutic areas on a common scale [22]. At the same time, DALYs do not capture all ethically or practically relevant considerations for priority-setting, and they should therefore be viewed as a practical proxy rather than a comprehensive measure of social value. We discuss the limitations of DALYs, and how they might be complemented by additional criteria, in Section 4.1.

By combining these three components, we formulate the optimal megafund portfolio construction problem as the following objective function:


$$\begin{array}{cc} \max_{w}\; & \lambda_{1}w^{⊤}\mu -\lambda_{2}w^{⊤}\Sigma w+\lambda_{3}w^{⊤}h,\\ \text{subject to}\; & \left\{ \begin{array}{c} w_{\text{low}}\leq w_{i}\leq w_{\text{high}},\;i=1,2,\ldots ,32,\\ w_{1}+w_{2}+\ldots +w_{32}=1. \end{array}\right. \end{array}$$ (5)

The constants *w*_low_ and *w*_high_ denote the minimum and maximum allowable weights for each category, ensuring that the portfolio is not overly concentrated in some areas while neglecting others. In our simulation, we set


$$\begin{array}{c} w_{\text{low}}=\frac{1}{32}-0.02,\;w_{\text{high}}=\frac{1}{32}+0.02, \end{array}$$

which allows each weight to deviate by at most 2% from the equal-weighted portfolio.

The parameters $\lambda_{1},\lambda_{2},\lambda_{3}\geq 0$ are tuning coefficients that reflect the relative importance of expected return, variance, and healthcare impact in the objective function. If $\lambda_{1}=1$ and $\lambda_{2}=\lambda_{3}=0$, the problem reduces to maximizing expected return (Eq (2)); if $\lambda_{2}=1$ and $\lambda_{1}=\lambda_{3}=0$, it minimizes portfolio risk (Eq (3)); and if $\lambda_{3}=1$ and $\lambda_{1}=\lambda_{2}=0$, it maximizes healthcare impact (Eq (4)). If we set only $\lambda_{3}=0$, the healthcare impact is ignored, and the problem reduces to the traditional mean–variance analysis framework in portfolio theory. In practice, investors may choose these three parameters according to their preference.

Although the optimization framework does not explicitly introduce biological feasibility or scientific maturity as separate constraints, these dimensions are indirectly reflected in several of the model parameters. In particular, areas that are scientifically more mature or tractable will tend to have higher probabilities of approval, *p*_*i*_, shorter development times, *T*_*i*_, and more favorable cost–value profiles, (*V*_*i*_, *X*_*i*_), while scientifically challenging or less feasible areas will be characterized by lower *p*_*i*_, longer *T*_*i*_, and less attractive economics. Through Eq (1), these differences translate into lower expected PIs, $\mu_{i}$, and higher variances, $\sigma_{i}^{2}$, for categories with poor technical viability, which in turn reduce the optimal weight, *w*_*i*_.

At the same time, healthcare impact enters through *h*_*i*_, which reflects both the DALY burden and the expected cost of a successful project in each category. In our implementation, we do not impose hard DALY thresholds; instead, *h*_*i*_ enters continuously in the healthcare-impact term of the objective function, so areas with greater and more cost-tractable disease burden receive higher impact scores. The objective function in Eq (5) therefore balances impact-driven investment against technical and commercial viability: categories with high disease burden but very low PoS or unfavorable cost–value profiles will only receive substantial weight if investors place a sufficiently large emphasis on healthcare impact (via $\lambda_{3}$) relative to expected return and risk (via $\lambda_{1}$ and $\lambda_{2}$). In practice, an HNF-like vehicle could further sharpen this balance by overlaying expert judgment or quantitative feasibility scores on top of the stage–area parameters—for example, by restricting or down-weighting categories with extremely low scientific tractability—so that impact-oriented allocation operates conditional on a minimum level of technical viability.

The optimization problem in Eq (5) is a standard quadratic programming problem that can be efficiently solved using methods such as the interior-point algorithm. In our simulations, we employ Python’s cvxpy package to solve the problem.

### 2.3 Simulation method

In this section, we introduce the simulation method behind our HNF, which builds on the licensing model commonly used in the biopharmaceutical industry [8] and extends prior studies [11–15].

**Fig 1:**
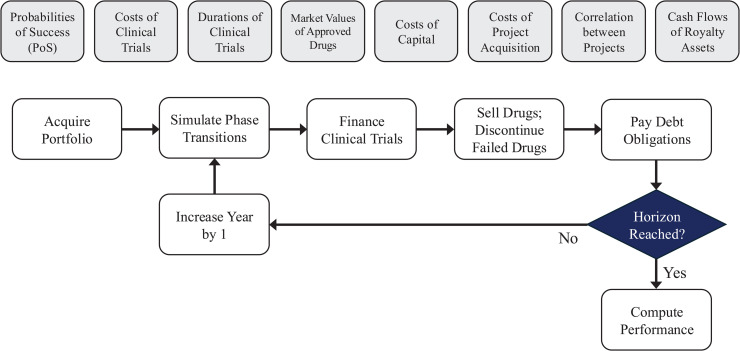
Flowchart of the megafund simulation framework. The gray boxes at the top represent the estimated parameters required for the simulation.

Fig 1 shows the simulation framework of our HNF. The input parameters of the framework are shown in gray boxes, including the PoS, cost of capital, duration, and cost of clinical trials, market values of approved drugs, and correlations across projects. The specific parameter values used in our simulation are detailed in S1 Text, and all values are adjusted to 2025 dollars. For simplicity, we assume that drug development projects within the same stage–area category share identical parameters, while parameters differ across categories.

As shown in Fig 1, the first step of our simulation is to acquire all assets in the portfolio. In particular, we first assign portfolio weights to each stage–area category according to the asset allocation strategy described in Sections 2.1 and 2.2. Next, for each category, we convert the allocated capital into the number of projects by dividing the total investment in that category by the acquisition cost of an individual project. Since the resulting number of projects must be an integer, we round to the nearest whole number and require at least one project in each category to maintain diversification. This may cause the actual fund size to deviate from the target ($12.5 billion). This procedure determines the number of projects acquired in each category and thereby defines the overall portfolio composition.

To estimate the fair acquisition value of a single project, we first obtain the market values of approved drugs in each therapeutic area. Then, for each project, we derive its value at earlier development stages using the following backward recursive relation:


$$\begin{array}{c} V_{x}=\max\left\{\frac{p_{x}V_{x+1}}{(1+r_{x}{)}^{t_{x}}}-C_{x},0\right\}, \end{array}$$ (6)

where *x* and *x* + 1 denote any stage and its subsequent stage of development, including preclinical, phase 1, phase 2, phase 3, New Drug Application (NDA), and regulatory approval. The parameters *V*_*x*_, *r*_*x*_, *t*_*x*_, and *C*_*x*_ represent the value, cost of capital, duration, and clinical trial cost of the project at stage *x*, respectively, and *p*_*x*_ is the probability that the project successfully transitions from stage *x* to *x* + 1.

The intuition behind Eq (6) is as follows. The expected revenue at stage *x* is given by *p*_*x*_*V*_*x*+1_, the PoS multiplied by the value at stage *x* + 1. The net present value of the project at stage *x* is therefore the present value of this expected revenue minus the trial cost, i.e., $p_{x}V_{x+1}/(1+r_{x}{)}^{t_{x}}-C_{x}$. We then take the maximum of this value and zero because, if the net present value is negative, the project manager would rationally choose to abandon the project to avoid losses. This decision rule is consistent with the real-options approach to clinical trials [23]. In our simulation, we use *C*_*x*_ as an estimate of the acquisition cost of the project at stage *x*.

After acquiring all assets in the portfolio, we simulate its evolution over the entire investment horizon, as illustrated in Fig 1. Projects that succeed at a given stage advance to the next, while those that fail are terminated. Each stage requires funding to cover clinical trial expenses, which is provided by the megafund. Once a project reaches regulatory approval, it is sold to generate revenue, and we assume a one-year lag between regulatory approval and asset sale. Revenues from successful exits, together with royalty income, constitute the cash flows used to service debt obligations.

At the end of each year, in accordance with the capital structure described in Section 2.1, proceeds are allocated first to senior debt holders, then to junior debt holders, and finally to equity holders. When the fund reaches the end of its investment horizon, it is liquidated. If at any time the fund is unable to meet its debt obligations, early liquidation is triggered. Following this simulation, we compute the financial performance separately for senior debt holders, junior debt holders, and equity holders.

## 3. Results

In this section, we conduct Monte Carlo simulations to evaluate the performance of our megafund portfolio. Section 3.1 presents the performance of the equal-weighted portfolio, which serves as the benchmark. Section 3.2 reports the performance of portfolios constructed using our optimization framework described in Section 2.2. Section 3.3 provides the sensitivity analysis. Each scenario is simulated 50,000 times.

### 3.1 Benchmark portfolio

We first evaluate the performance of the equal-weighted portfolio, which allocates investment evenly across clinical trial phases and therapeutic areas. This portfolio serves as a benchmark for assessing the benefits of our optimization framework.

**Fig 2:**
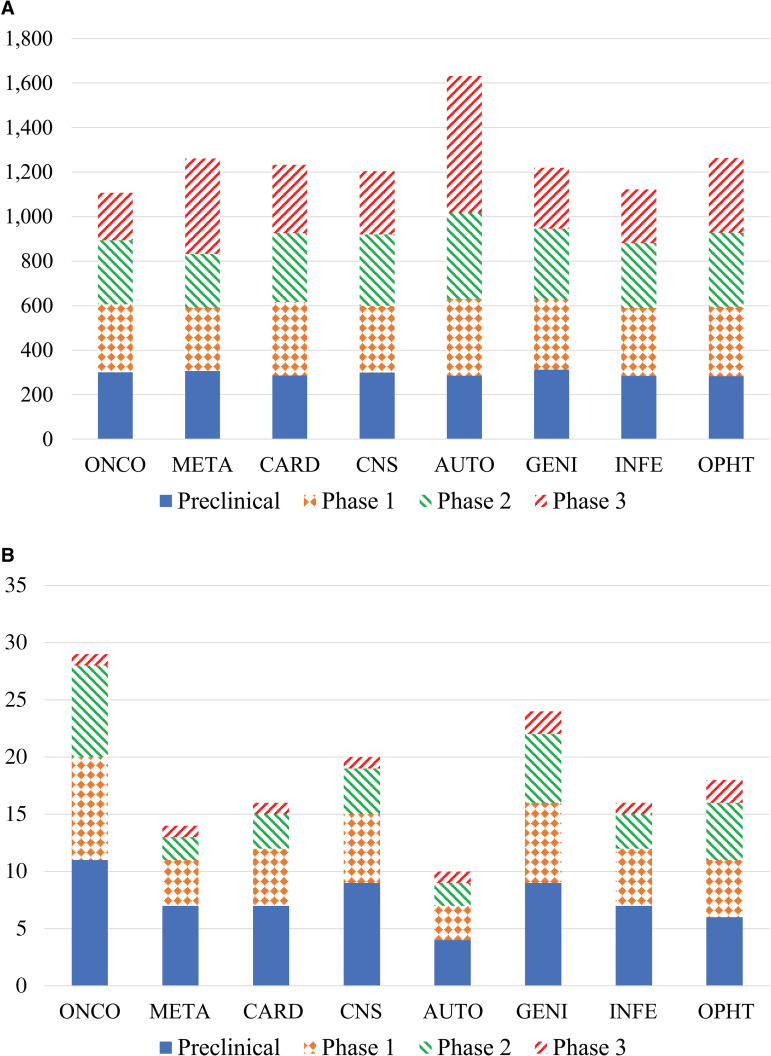
Expected investment and number of projects in the benchmark portfolio across therapeutic areas and clinical trial phases. Therapeutic areas are abbreviated as follows: ONCO (oncology), META (metabolic/endocrinology), CARD (cardiovascular), CNS (central nervous system), AUTO (autoimmune/inflammation), GENI (genitourinary), INFE (infectious disease), and OPHT (ophthalmology). (A) Expected investment ($ million) on each stage–area category. (B) Expected number of projects for each stage–area category.

Fig 2 illustrates the allocation of the benchmark portfolio across therapeutic areas and clinical trial stages. Fig 2A reports the expected investment amounts, while Fig 2B shows the corresponding expected number of projects. Each phase of development receives roughly $2.5 billion in investment, leading to a balanced allocation across therapeutic areas. Because preclinical projects have substantially lower acquisition costs compared to later-stage projects, the portfolio naturally contains a larger number of preclinical assets. This structure highlights the benchmark portfolio’s emphasis on diversification across both development stages and therapeutic areas.

The first column of Table 2 presents the performance statistics of the equal-weighted benchmark portfolio. For both the senior and junior debt tranches, we present the probability of default and the expected loss. A tranche is considered to be in default if any outstanding debt remains unpaid at the end of the investment horizon. Expected loss is calculated by dividing the unpaid debt by the total debt issued. The probabilities of default for the senior and junior tranches are 0.030% and 0.116%, respectively, which are broadly comparable to those of AAA- and AA-rated bonds, respectively.

**Table 2:** Performance measures of the equal-weighted benchmark portfolio and the four portfolios optimized under Eq (5): the Mean Portfolio, Variance Portfolio, Mean–Variance Portfolio, and Healthcare Portfolio.

Portfolio Type	Benchmark	Mean	Variance	Mean–Variance	Healthcare
**Capital Structure**					
Total ($ million)	12,500	13,600	12,300	13,600	13,300
Senior Debt ($ million)	7,500	8,160	7,380	8,160	7,980
Junior Debt ($ million)	1,250	1,360	1,230	1,360	1,330
Equity ($ million)	3,750	4,080	3,690	4,080	3,990
Tenor (years)	14.5	14.5	14.5	14.5	14.5
**Senior Debt**					
Prob. of Default (bp)	3.0	2.0	1.6	2.4	1.0
Expected Loss (bp)	0.3	0.2	0.2	0.2	0.1
**Junior Debt**					
Prob. of Default (bp)	11.6	14.6	8.2	9.6	10.2
Expected Loss (bp)	6.5	8.3	3.9	5.2	5.3
**Equity**					
Mean of Ann. ROE (%)	12.0	**12.5**	11.7	12.5	12.4
SD of Ann. ROE (%)	5.9	6.2	**5.1**	5.5	5.5
Ann. Sharpe Ratio	1.37	1.37	1.49	**1.54**	1.51
Prob. of Wipeout (bp)	13.4	15.6	**9.0**	10.6	10.8
Prob. of Loss (%)	1.5	1.4	1.4	**1.2**	1.3
Prob. of Ann. ROE ≥ 10% (%)	75.2	77.7	74.2	**78.2**	77.3
**Expected Approvals**					
Oncology	1.8	1.9	1.4	1.9	3.1
Metabolic/Endocrinology	2.0	2.9	1.5	2.9	1.1
Cardiovascular	2.8	3.7	2.3	3.6	4.4
Central Nervous System	2.8	4.3	2.0	3.1	4.2
Autoimmune/Inflammation	1.5	1.7	1.5	1.8	1.0
Genitourinary	6.1	2.2	9.8	4.3	4.1
Infectious Disease	4.6	7.0	5.8	6.9	7.8
Ophthalmology	3.1	1.2	3.1	1.2	1.3
Total	24.7	24.8	27.4	25.6	26.9
**Healthcare Impact**					
Affected Prevalence (million)	44.0	43.9	48.6	45.2	40.8
Affected DALYs (million)	243.9	296.7	241.7	288.5	**325.3**

The bolded values indicate the best-performing measure across portfolios.

For equity investors, Table 2 reports the mean and standard deviation (SD) of the annualized return on equity (ROE) across all simulations, as well as the annualized Sharpe ratio. The Sharpe ratio is a standard measure of risk-adjusted portfolio performance in finance and is defined as


$$\begin{array}{c} \text{Sharpe ratio}=\frac{𝔼[ROE]-r_{f}}{SD(ROE)}, \end{array}$$

where *r*_*f*_ is the risk-free rate (set to 4.0%), and 𝔼[ROE] and SD(ROE) are the mean and SD of the annualized ROE. Intuitively, the Sharpe ratio measures the excess return per unit of risk (volatility), so higher values indicate more attractive risk-adjusted performance. The equal-weighted benchmark portfolio delivers an expected annualized ROE of 12.0%—broadly comparable to the historical annualized return of the S&P 500 from 2010 to 2024—with a Sharpe ratio of 1.37.

Table 2 also reports the probabilities of wipeout, loss, and achieving an annualized ROE greater than 10% for equity investors. Here, wipeout refers to scenarios in which equity investors lose their entire investment, and loss refers to scenarios with a negative annualized ROE. The probability of wipeout is extremely low at 0.134%, the probability of loss is 1.5%, and the probability of earning an annualized ROE above 10% is approximately 75.2%. Together, these results highlight the robustness of the megafund strategy, demonstrating its ability to generate attractive financial returns.

In addition to financial performance, Table 2 also reports the average number of drug approvals across the different therapeutic areas as well as the healthcare impact of the megafund. On average, the equal-weighted benchmark portfolio supports 24.7 approvals, with relatively higher concentrations in the genitourinary, infectious disease, and ophthalmology areas. These approved drugs are expected to benefit approximately 44.0 million patients worldwide and address an estimated 243.9 million DALYs, underscoring the fund’s substantial global health impact.

Taken together, these results demonstrate that the HNF is capable of delivering attractive financial returns to both debt and equity investors while simultaneously generating significant social value, even under a simple equal-weighted allocation. This dual capacity makes the megafund a compelling vehicle not only for private investors but also for institutions ranging from philanthropic organizations to governments seeking to advance global public health outcomes. In the next section, we examine how the optimization framework introduced in Section 2.2 can further improve both the financial performance and the healthcare impact of the megafund.

### 3.2 Optimal portfolios

In this section, we analyze the performance of various portfolios optimized according to Eq (5). By tuning the parameters in the objective function, $\lambda_{1}$, $\lambda_{2}$, and $\lambda_{3}$, we construct the following portfolios and compare their performance against the equal-weighted benchmark portfolio (Section 3.1):

- the mean portfolio ($\lambda_{1}=1$ and $\lambda_{2}=\lambda_{3}=0$), which maximizes the expected return;
- the variance portfolio ($\lambda_{2}=1$ and $\lambda_{1}=\lambda_{3}=0$), which minimizes the risk;
- the mean–variance portfolio ($\lambda_{1}=2$, $\lambda_{2}=0.1$, and $\lambda_{3}=0$), which balances the return and risk;
- the healthcare portfolio ($\lambda_{3}=1$ and $\lambda_{1}=\lambda_{2}=0$), which maximizes the healthcare impact.

The last four columns of Table 2 show the performance metrics for these portfolios, revealing clear trade-offs aligned with each portfolio’s objective. First, the mean portfolio, which focuses exclusively on maximizing the expected return, achieves the highest annualized ROE (12.5%). Second, the variance portfolio has the smallest SD of annualized ROE (5.1%) and probability of wipeout (0.09%).

Third, the mean–variance portfolio demonstrates the value of incorporating risk into the optimization. By balancing the maximization of mean return with the minimization of variance, this portfolio yields the highest annualized Sharpe ratio (1.54), indicating the most efficient risk-adjusted returns. Fourth, the healthcare portfolio, which is structured to maximize healthcare impact, scores the highest on metrics of social benefit—it is projected to generate the greatest value in terms of affected DALYs (325.3 million). In other words, with a total investment of $13.3 billion, the healthcare portfolio is expected to save 325.3 million life years, which represents a substantial impact on society. The $13.3 billion fund size is slightly above the $12.5 billion target because of the integer rounding and diversification constraints discussed in Section 2.3.

Finally, by comparing the performance of the four portfolios with that of the equal-weighted benchmark portfolio, we observe that all four optimal portfolios outperform the benchmark in terms of Sharpe ratio, probability of loss, and expected number of project approvals, in addition to each outperforming the benchmark in other aspects, thanks to their distinct objective focuses. These results further highlight that, although the equal-weighted benchmark megafund already delivers attractive returns to investors, its performance can be enhanced through the optimization framework given in Eq (5).

**Fig 3:**
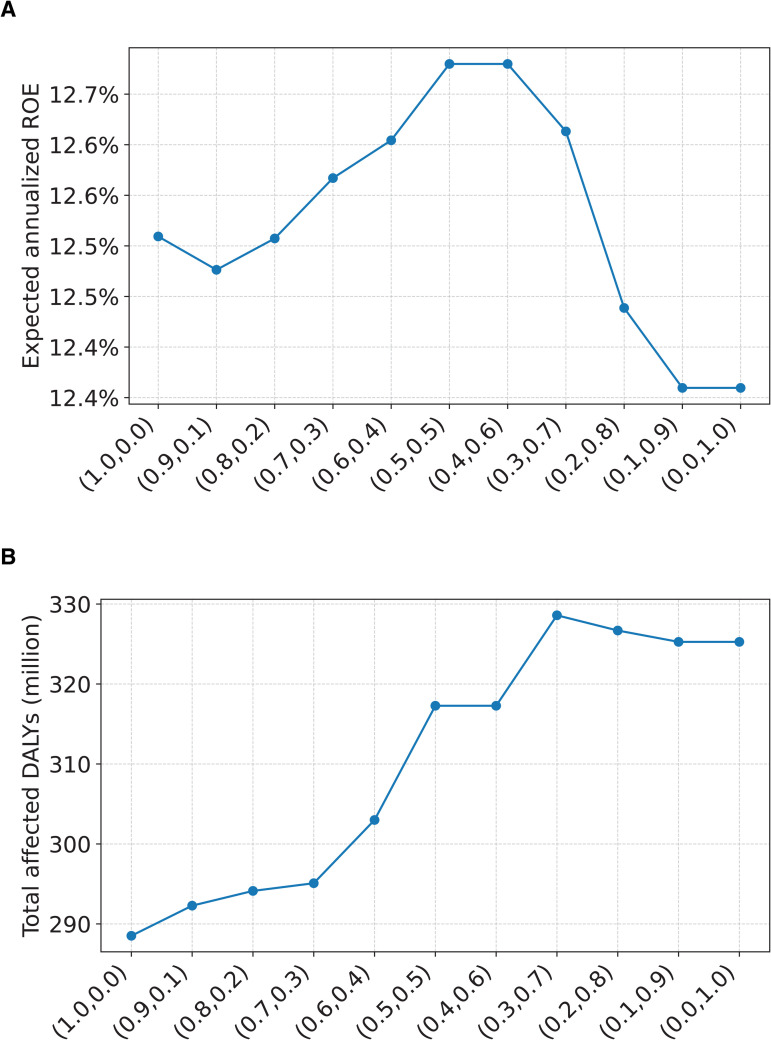
Expected annualized ROE and healthcare impacts of the optimized portfolios as investor preference shifts from financial return ($\lambda_{1}\;$) to healthcare impact ($\lambda_{3}\;$), with $\lambda_{2}\;$ held constant at 0.1. The *x*-axis labels denote the relative preference pair for financial performance and healthcare impact, ranging from (1.0, 0.0) (the mean–variance portfolio) to (0.0, 1.0) (the healthcare portfolio). Fig 3A shows the expected annualized ROE for each portfolio. Fig 3B presents the corresponding healthcare impact, computed as the sum of approved projects in each therapeutic area weighted by their respective DALYs. (A) Expected annualized ROE. (B) Expected healthcare impact.

We further study how the financial performance and the healthcare impact of the optimal portfolio constructed using Eq (5) will vary with respect to the parameters characterizing investor preference: $\lambda_{1}$, $\lambda_{2}$, and $\lambda_{3}$. In Fig 3, we present the expected annualized ROE and healthcare impact as investor preference shifts from a focus on financial return ($\lambda_{1}$) to healthcare impact ($\lambda_{3}$), while keeping the preference for variance minimization constant ($\lambda_{2}=0.1$). We set the ranges of $\lambda_{1}$ and $\lambda_{3}$ to $\lambda_{1}\in [0,2]$ and $\lambda_{3}\in [0,50]$, respectively, because the magnitudes of the three components in Eq (5)—Eqs (2), (3), and (4)—are different. We normalize them to give their values similar orders of magnitude, and also to avoid any term dominating the objective function. The *x*-axis in Fig 3 represents the relative proportion between $\lambda_{1}$ and $\lambda_{3}$. For example, an *x*-axis value of (0.2, 0.8) corresponds to $\lambda_{1}=0.2\times 2=0.4$ and $\lambda_{3}=0.8\times 50=40$. By changing this relative proportion pair from (1.0, 0.0) to (0.0, 1.0), the investor preference shifts from a sole focus on financial performance to a sole focus on healthcare. In particular, when the pair takes the values (1.0, 0.0) and (0.0, 1.0), the portfolio is close to the mean–variance portfolio and the healthcare portfolio described earlier, respectively.

Fig 3 shows the trade-offs between the financial performance and the healthcare impact of the portfolio: increasing $\lambda_{3}$ relative to $\lambda_{1}$ shifts the portfolio toward a higher DALY-weighted healthcare impact at the expense of lower expected equity returns, while the reverse weighting achieves the opposite. However, these trade-offs are not strictly unidirectional, as the inclusion of a nonzero $\lambda_{2}$ term ensures that variance reduction remains a secondary consideration across all portfolios, slightly moderating both return and healthcare outcomes.

### 3.3 Sensitivity analysis

Given the complexity of the HNF and the large number of parameters involved, we conduct a sensitivity analysis on the key inputs of the megafund model to assess performance under different conditions. Sections 3.3.1, 3.3.2, 3.3.3, and 3.3.4 analyze the effects of the debt-to-equity leverage ratio, the PoS, the market value of developed drugs, and the cost of capital used for project valuation in turn. For consistency and comparability, this section focuses on the equal-weighted benchmark portfolio.

#### 3.3.1 Leverage.

The simulation results in Section 3.1 are based on a leveraged capital structure with senior debt, junior debt, and equity in a 60:10:30 ratio. To evaluate how different leverage ratios affect portfolio performance, this section focuses solely on senior debt, excluding junior tranches, in order to isolate the impact of high-priority obligations. The coupon rate of the senior debt is set as 5%. Table 3 shows the performance of the equal-weighted benchmark megafund under different debt-to-equity ratios—80:20, 60:40, 40:60, 20:80, and 0:100.

**Table 3:** Performance measures of the benchmark megafund portfolio under varying debt-to-equity leverage ratios.

Debt-to-Equity Ratio	80:20	60:40	40:60	20:80	0:100
**Capital Structure**					
Total ($ million)	12,500	12,500	12,500	12,500	12,500
Debt ($ million)	10,000	7,500	5,000	2,500	0
Equity ($ million)	2,500	5,000	7,500	10,000	12,500
Tenor (Years)	14.5	14.5	14.5	14.5	14.5
**Debt**					
Prob. of Default (bp)	43.6	1.0	0.0	0.0	0.0
Expected Loss (bp)	4.3	0.1	0.0	0.0	0.0
**Equity**					
Mean of Ann. ROE (%)	13.9	11.7	10.2	9.2	8.4
SD of Ann. ROE (%)	9.1	3.6	2.6	2.1	1.9
Ann. Sharpe Ratio	1.09	2.16	2.40	2.41	2.38
Prob. of Wipeout (bp)	44.8	1.2	0.0	0.0	0.0
Prob. of Loss (%)	1.8	0.5	0.2	0.1	0.0
Prob. of Ann. ROE ≥ 10% (%)	84.2	73.6	56.3	36.9	20.9
**Expected Approvals**					
Oncology	1.6	2.4	2.9	3.0	3.0
Metabolic/Endocrinology	2.0	2.5	2.6	2.6	2.6
Cardiovascular	2.4	3.2	3.3	3.3	3.3
Central Nervous System	2.8	3.2	3.2	3.2	3.2
Autoimmune/Inflammation	1.6	2.2	2.4	2.4	2.4
Genitourinary	6.0	6.2	6.2	6.2	6.2
Infectious Disease	4.2	4.7	4.8	4.8	4.8
Ophthalmology	2.9	3.3	3.4	3.4	3.4
Total	23.4	27.8	28.9	28.9	28.9

The bolded values indicate the best-performing measure across portfolios.

We observe that, as the leverage ratio increases (i.e., a higher proportion of debt in the capital structure), the probability of default on the debt also increases. This outcome is intuitive, since higher leverage requires larger and more frequent cash outflows to service coupon payments, leaving the fund more vulnerable to shortfalls in operating cash flows. Conversely, reducing the leverage ratio decreases the probability of default, enhancing the overall financial stability of the fund. However, this greater security comes at the cost of potentially lower returns to equity investors, since less debt financing limits the degree of financial amplification.

Notably, the highest annualized ROE (13.9%) is achieved by the most highly leveraged megafund with an 80:20 debt-to-equity ratio. In contrast, the three low-leverage megafunds (with 40:60, 20:80, and 0:100 ratios) achieve the highest expected number of drug approvals (28.9). This highlights the megafund’s capacity to carry all projects to completion when facing little risk of default, thereby maximizing the portfolio’s effectiveness in developing approved drugs. In this sense, the low-leverage portfolios trade potential financial upside for a more reliable contribution to public health outcomes.

These findings also emphasize the trade-offs between leverage and portfolio performance. Higher leverage can significantly boost returns to equity investors, but it also escalates exposure to default risk. Lower leverage, by contrast, offers a more stable and socially effective strategy, though at the expense of reduced financial returns.

#### 3.3.2 Probability of successful phase transition.

The PoS in clinical trials is a key parameter that reflects the likelihood of a drug candidate progressing from the preclinical stage through phases 1, 2, and 3 to eventual approval. The simulation results in Section 3 are derived from the baseline PoSs estimated from historical data and a review of the literature (see Table A in S1 Text). To evaluate the impact of changes in these probabilities on portfolio performance, in this section, we scale the baseline values to 90%, 95%, 105%, and 110% of their original values across all phases. Table 4 reports the performance of the equal-weighted benchmark megafund under these alternative PoS scenarios.

**Table 4:** Performance measures of the benchmark megafund portfolio under varying probabilities of successful phase transition.

PoS	Baseline	90% PoS	95% PoS	105% PoS	110% PoS
**Capital Structure**					
Total ($ million)	12,500	12,300	12,200	12,500	12,500
Senior Debt ($ million)	7,500	7,380	7,320	7,500	7,500
Junior Debt ($ million)	1,250	1,230	1,220	1,250	1,250
Equity ($ million)	3,750	3,690	3,660	3,750	3,750
Tenor (Years)	14.5	14.5	14.5	14.5	14.5
**Senior Debt**					
Prob. of Default (bp)	3.0	4.8	2.2	0.6	0.8
Expected Loss (bp)	0.3	0.4	0.3	0.1	0.0
**Junior Debt**					
Prob. of Default (bp)	11.6	33.8	19.8	3.6	3.0
Expected Loss (bp)	6.5	17.7	10.3	1.5	1.8
**Equity**					
Mean of Ann. ROE (%)	12.0	10.3	11.3	12.7	13.1
SD of Ann. ROE (%)	5.9	8.3	6.9	4.5	4.1
Ann. Sharpe Ratio	1.37	0.76	1.06	1.94	2.25
Prob. of Wipeout (bp)	13.4	35.4	21.8	4.2	3.4
Prob. of Loss (%)	1.5	4.0	2.4	0.9	0.6
Prob. of Ann. ROE ≥ 10% (%)	75.2	62.9	69.5	80.2	84.4
**Expected Approvals**					
Oncology	1.8	1.6	1.7	2.0	2.1
Metabolic/Endocrinology	2.0	1.8	2.0	2.0	2.3
Cardiovascular	2.8	2.5	2.4	3.1	2.9
Central Nervous System	2.8	2.3	2.5	2.9	3.1
Autoimmune/Inflammation	1.5	1.4	1.4	1.5	1.8
Genitourinary	6.1	4.7	5.1	6.4	6.6
Infectious Disease	4.6	4.5	4.6	5.0	5.1
Ophthalmology	3.1	2.3	2.6	3.6	3.2
Total	24.7	21.1	22.4	26.5	27.1

Table 4 demonstrates that increasing the PoS leads to a notable improvement in the portfolio’s performance. Specifically, the number of approved drugs rises, with the scenario with the highest PoS increase yielding 27.1 approvals compared to the baseline approval rate of 24.7. Moreover, a higher PoS increases the average ROE and reduces volatility, thereby improving the Sharpe ratio. For instance, when the PoS is set at 110% of the baseline, the megafund achieves a Sharpe ratio of 2.25, far higher than the baseline value of 1.37. Even when the PoS is reduced to 90% of the baseline, however, the portfolio still delivers a double-digit annualized ROE (10.3%) while keeping the probability of default low for both the senior and junior tranches (0.048% and 0.338%, respectively).

These findings suggest that by choosing drug development projects with a potentially higher PoS, the performance of the fund can be significantly improved. In particular, practitioners may use advanced machine learning methods to predict clinical trial outcomes [24] and thereby invest in projects with higher PoS. Leveraging expert selection processes and predictive technologies to enhance PoS may lead to substantial gains in both the number of approved drugs and the financial returns of the megafund.

#### 3.3.3 Market value of drugs.

The market value of approved drugs is a central determinant of the megafund’s financial performance, as it directly shapes the magnitude of potential returns. The simulation results in Section 3 use the baseline market values of approved drugs obtained from market research reports (see Table D in S1 Text). To evaluate the impact of varying these market values on portfolio performance, in this section, we consider alternative scenarios in which the market value of drugs is scaled to 50%, 75%, 150%, and 200% of the baseline. Table 5 reports the performance of the equal-weighted benchmark megafund under these scenarios.

**Table 5:** Performance measures of the benchmark megafund portfolio under varying market values of approved drugs.

Market Value (MV)	Baseline	50% MV	75% MV	150% MV	200% MV
**Capital Structure**					
Total ($ million)	12,500	12,000	12,000	13,400	14,300
Senior Debt ($ million)	7,500	7,200	7,200	8,040	8,580
Junior Debt ($ million)	1,250	1,200	1,200	1,340	1,430
Equity ($ million)	3,750	3,600	3,600	4,020	4,290
Tenor (Years)	14.5	14.5	14.5	14.5	14.5
**Senior Debt**					
Prob. of Default (bp)	3.0	1.4	0.4	5.2	13.2
Expected Loss (bp)	0.3	0.1	0.0	0.4	1.6
**Junior Debt**					
Prob. of Default (bp)	11.6	19.0	9.2	20.0	41.0
Expected Loss (bp)	6.5	7.9	3.0	12.5	26.6
**Equity**					
Mean of Ann. ROE (%)	12.0	9.0	11.1	12.5	12.0
SD of Ann. ROE (%)	5.9	6.6	5.4	6.8	8.8
Ann. Sharpe Ratio	1.37	0.75	1.32	1.24	0.91
Prob. of Wipeout (bp)	13.4	21.8	9.8	20.8	43.4
Prob. of Loss (%)	1.5	3.4	1.7	1.8	2.7
Prob. of Ann. ROE ≥ 10% (%)	75.2	48.3	67.8	78.0	76.1
**Expected Approvals**					
Oncology	1.8	2.2	2.1	1.2	1.0
Metabolic/Endocrinology	2.0	3.0	2.4	1.8	1.3
Cardiovascular	2.8	4.5	3.3	1.9	1.6
Central Nervous System	2.8	3.9	3.1	2.2	1.7
Autoimmune/Inflammation	1.5	1.7	1.5	1.3	1.0
Genitourinary	6.1	7.2	6.7	4.5	3.5
Infectious Disease	4.6	6.8	6.1	3.6	3.0
Ophthalmology	3.1	3.9	3.1	2.3	1.7
Total	24.7	33.2	28.3	18.7	14.7

Table 5 shows that, when the market value of the drugs decreases, the financial performance of the megafund deteriorates. For example, at 50% of the baseline market value, the expected annual ROE drops to 9.0%, with a Sharpe ratio of only 0.75, compared to 12.0% and 1.37 at the baseline. However, the lower market value also reduces the acquisition cost for each project, allowing the megafund to acquire more drug development projects in the portfolio. As a result, the expected number of approvals increases to 33.2 compared to 24.7 at the baseline. This expansion of the portfolio implies that, although financial returns may be lower, the social impact is potentially larger due to a higher number of approved drugs.

Conversely, while moderate increases in market value can slightly enhance financial returns, excessively high market values reduce the number of projects that can be acquired, thereby undermining diversification. For instance, when market values double relative to the baseline, the expected annualized ROE remains at 12.0%, but the Sharpe ratio declines markedly from 1.37 to 0.91, and the probability of default on senior and junior debt rises to 0.132% and 0.410%, respectively. At the same time, the total number of approved drugs falls to 14.7 compared with 24.7 under the baseline scenario, demonstrating that reduced diversification will weaken both financial stability and the healthcare impact of the megafund.

These findings highlight the importance of balancing drug market value with portfolio size and costs. While low market values undermine financial returns, they allow the inclusion of more projects, increasing the total number of approvals. Conversely, excessively high market values limit the number of projects in the portfolio, increasing risk and volatility. Managing this balance is essential to achieving stable and high-performing megafund outcomes, including optimizing the Sharpe ratio and maintaining a low default risk.

#### 3.3.4 Cost of capital for project valuation.

The cost of capital applied to project valuation in the megafund portfolio is an important determinant because it directly affects the acquisition cost of each project when calculating net present value in Eq (6). A higher cost of capital reduces the acquisition price, providing greater financial advantage to the buyer—in this case, the megafund. The baseline cost of capital used in Section 3 is estimated from Avance’s survey of discount rates in biotechnology [25] and adjusted to reflect 2024 conditions by adding 5.25%, corresponding to the change in the U.S. policy rate between May 2021 and August 2024 (see Table E in S1 Text).

To evaluate the impact of varying costs of capital on portfolio performance in this section, we scale the baseline value downward by 1.75%, 3.50%, and 5.25%, and compare the outcomes to the baseline case. Table 6 summarizes the performance of the equal-weighted benchmark megafund under these alternative costs of capital.

**Table 6:** Performance measures of the benchmark megafund portfolio under varying costs of capital for project valuation.

Cost of Capital (CoC)	Baseline	CoC − 5.25%	CoC − 3.50%	CoC − 1.75%
**Capital Structure**				
Total ($ million)	12,500	12,700	12,700	12,400
Senior Debt ($ million)	7,500	7,620	7,620	7,440
Junior Debt ($ million)	1,250	1,270	1,270	1,240
Equity ($ million)	3,750	3,810	3,810	3,720
Tenor (Years)	14.5	14.5	14.5	14.5
**Senior Debt**				
Prob. of Default (bp)	3.0	9.0	5.0	3.0
Expected Loss (bp)	0.3	0.6	0.5	0.3
**Junior Debt**				
Prob. of Default (bp)	11.6	46.2	26.6	20.8
Expected Loss (bp)	6.5	25.6	14.3	10.4
**Equity**				
Mean of Ann. ROE (%)	12.0	8.6	10.0	11.0
SD of Ann. ROE (%)	5.9	9.0	7.4	6.8
Ann. Sharpe Ratio	1.37	0.52	0.80	1.04
Prob. of Wipeout (bp)	13.4	49.2	28.2	21.6
Prob. of Loss (%)	1.5	5.0	3.4	2.3
Prob. of Ann. ROE ≥ 10% (%)	75.2	49.3	59.3	68.2
**Expected Approvals**				
Oncology	1.8	1.3	1.5	1.7
Metabolic/Endocrinology	2.0	1.5	1.7	1.8
Cardiovascular	2.8	1.7	2.1	2.4
Central Nervous System	2.8	2.1	2.3	2.5
Autoimmune/Inflammation	1.5	1.1	1.1	1.2
Genitourinary	6.1	5.2	5.7	5.7
Infectious Disease	4.6	3.5	3.9	4.5
Ophthalmology	3.1	2.7	2.9	2.9
Total	24.7	19.0	21.1	22.8

As the cost of capital decreases by 5.25% from the 2024-adjusted baseline to the 2021 level, the fund’s performance weakens, with the average annualized ROE falling from 12.0% to 8.6%, and the Sharpe ratio dropping from 1.37 to 0.52. Conversely, as the cost of capital rises, the acquisition costs decline, allowing the megafund to support more projects, thus improving both financial and social outcomes. For example, at the baseline cost of capital, the expected number of drug approvals is 24.7, compared to only 19.0 under the 2021 cost of capital. Similarly, the probability of achieving an annualized ROE above 10% increases steadily from 49.3% at the 2021 cost of capital to 75.2% at the baseline level. Despite these differences, all scenarios except the 2021 cost of capital still deliver double-digit annualized ROE. Note that, in practice, the cost of capital is tied to the prevailing risk-free rate and coupon obligations; when interest rates fall, debt servicing becomes less costly, suggesting that actual performance may be stronger than what is reported in Table 6.

## 4. Discussion

In this section, we discuss the limitations and practical considerations of our HNF framework in Sections 4.1 and 4.2, respectively.

### 4.1 Limitations

Our study has several limitations that point to promising directions for future work.

First, our analysis assumes that all drug development projects in the same therapeutic area share the same model parameters, thereby inevitably smoothing out heterogeneity across individual drug candidates. A logical next step would be to extend the model to the drug level when sufficiently granular project-level data are available, allowing for finer-grained optimization that accounts for these differences. This granularity may further improve both the financial efficiency of the megafund and its healthcare outcomes, especially when coupled with advanced predictive models for clinical trial success. In settings where such detailed data are not available, a related approach would be to specify distributions for key parameters within each stage–area category (such as PoS, costs, and market values) and sample project-specific draws from these distributions, thereby approximating candidate-level heterogeneity and producing a richer efficient frontier than the category-average simulations we report here.

Second, our calibration relies on historical averages for PoS, costs, development durations, and cash flows, implicitly assuming that past clinical development experience is a reasonable guide to future performance. This assumption will not hold exactly as science, regulation, trial design, and market conditions evolve, so our results are best interpreted as structured scenario analyses under a specific set of stylized assumptions rather than as point forecasts. Although these historical averages embed, in a coarse way, the biological feasibility and scientific maturity of different disease areas, the optimization framework does not explicitly represent these dimensions or allow them to directly constrain portfolio construction. In practice, an HNF-like vehicle would likely overlay expert judgment or quantitative feasibility or tractability scores at the project-selection stage, and extending the framework to incorporate such measures more directly is a natural direction for future work.

Third, our analysis assumes a static, buy-and-hold investment strategy: all clinical trial assets are acquired at the beginning of the fund’s life and held until success or failure, without explicit project-termination or reinvestment rules. This simplification improves the transparency and tractability of the present analysis but does not fully reflect how a real-world HNF would manage risk and capital over time. In practice, dynamic allocation strategies that respond to interim results over the investment horizon—such as staged go/no-go decisions, early termination of underperforming projects, and reinvestment of proceeds—could better accommodate real-world risk factors and, in expectation, improve risk-adjusted performance by both increasing returns and reducing downside risk. As data availability and predictive analytics improve, integrating such real-time information into the megafund portfolio construction and rebalancing is likely to become an important source of competitive advantage in drug-development financing. The static portfolios analyzed in this paper should therefore be interpreted as benchmark cases, providing a conservative baseline for what a dynamically managed HNF could achieve.

Finally, a further limitation concerns our use of DALYs as the primary summary measure of healthcare impact at the therapeutic-area level. DALYs are widely used to compare disease burdens across conditions and regions and offer a practical proxy for population health loss. However, burden alone does not reliably indicate scientific tractability, research feasibility, or commercial viability. For example, large burden-attention gaps have been documented in global health R&D [26,27], and DALYs often fail to align with research investment levels or scientific output [28,29]. Conceptual and methodological critiques further emphasize that DALYs do not capture whether effective biomedical interventions are technically achievable or readily translatable [30,31]. In this article, we therefore treat DALYs as one informative input into a multi-objective optimization problem rather than a stand-alone rule for investment prioritization. Moreover, there is an asymmetry between the specificity of clinical trials and approved indications and the breadth of conditions represented by DALY values: any one drug or approval will typically address only a small subset of the indications aggregated into a DALY estimate, so the realized benefit of a given product may be proportionately smaller or larger than the aggregated DALY value suggests. A more granular representation of disease burden would allow future extensions of the framework to track “addressable” health impact more directly and to assess how such refinements affect optimal portfolios.

### 4.2 Practical considerations

Translating the HNF concept into a real-world investment vehicle would raise several practical design and implementation questions.

An HNF would require a clearly defined governance and operating structure. Assets would likely be sourced through partnerships with established counterparties (pharmaceutical and biotechnology companies, and academic or non-profit product development partnerships), supported by regional networks that manage local regulatory, due diligence, and licensing requirements. Portfolio construction and asset-selection decisions would be made by a professional investment team, overseen by a global investment committee with expertise in both finance and drug development and informed by regional advisory boards familiar with local scientific, regulatory, and market conditions. To ensure transparency, accountability, and alignment with both financial and healthcare objectives, an HNF-like vehicle would also require independent oversight bodies (for example, a risk and audit committee and an impact/ethics committee with representation from investors, global health stakeholders, and independent experts) and regular public reporting on portfolio composition, financial performance, and health outcomes.

For the purposes of our simulations, we abstract from these institutional details and assume that, after such screening and due diligence, the projects available to the HNF have risk–return characteristics consistent with the stage–area averages in our input data, and we do not model information asymmetries between the fund and its counterparties. These governance and operational functions would inevitably entail management fees and other operating costs, so the returns reported in this article are best interpreted as gross of such expenses, with actual net performance depending on the specific fee and cost structure adopted by the fund.

In addition, for the sake of simplicity and to err on the side of conservative estimates of financial performance, our analysis has not explicitly modeled one of the biggest potential advantages of the size and global mandate of the HNF: the ability to attract nation-states as investors and offer them financial returns in excess of typical investment vehicles by addressing systemic country-level healthcare challenges. For example, consider a country X that seeks to develop manufacturing capabilities for producing mRNA vaccines of the type offered by Moderna and Pfizer–BioNTech during the COVID-19 pandemic. With sufficient scale and scientific/business credibility, the HNF should be able to negotiate attractive international partnerships in which country X invests a significant amount of capital directly—or indirectly via its sovereign wealth fund—in the HNF in exchange for:

- an agreement to open and operate parallel clinical trial sites in country X;
- a commitment to provide access to all HNF-approved drugs to country-X residents at a pre-negotiated price for a minimum number of doses over the first five years post-launch;
- and a commitment to invest in country X’s biomedical infrastructure to manufacture mRNA vaccines domestically if there is sufficient interest and capabilities to support such infrastructure building in country X.

Parallel trial sites will allow drug testing to yield valuable information about a drug candidate’s safety and efficacy profile across a greater diversity of patient populations. This information is especially valuable for countries populated by ethnic groups that are different than the typically Caucasian clinical trial participants in Western countries, where most new drugs are currently being developed. And advance purchase commitments are particularly welcomed by the biopharma sector because they can greatly reduce a drug’s launch risk, a key issue preventing most drug companies from investing in these markets. These methods allow the HNF to derisk participation for greater beneficial impact on health outcomes.

By investing in the creation of manufacturing infrastructure in country X, HNF investors enjoy improved risk-adjusted returns. But country X also benefits—not just from these infrastructure investments and HNF returns, but also from increases in employment, tax revenues, and access to cutting-edge medical technology, not to mention the positive network externalities of sharing in such biomedical innovation. This “virtuous cycle” yields tangible financial benefits for HNF investors, which in turn should attract a larger amount of capital in steady state.

By operating at a level higher than typical private-sector investment managers, the HNF creates greater value for nation-states and a portion of this incremental value will be shared with HNF investors in the form of higher risk-adjusted returns. This systemic view of the HNF underscores its potential in achieving global impact in an economically sustainable fashion that has so far eluded other private-sector business models.

One final practical consideration to discuss is the status of the original megafund structure, first proposed by Fernandez, Stein, and Lo [8] over a decade ago. Aspects of the model have been successfully implemented, especially the use of portfolio theory in de-risking a collection of therapeutic programs undertaken simultaneously rather than sequentially. This approach has been successfully demonstrated by BridgeBio Pharma [7,32], Roivant Sciences, and several other biotech companies [6]. Moreover, the successful multi-billion-dollar fundraises over the last decade by private-equity firms such as Blackstone, Carlyle, KKR, and others for investing in the life sciences and the broader healthcare sector are yet another manifestation of the megafund model. But a pure biomedical megafund has yet to be launched, perhaps because still missing from the ecosystem is a widely accepted set of metrics—including sophisticated default-probability models for debt that is collateralized by biomedical assets—for measuring the risk and reward of drug- and device-development programs, akin to the standardized metrics available for investments such as stocks, bonds, foreign currencies, commodities, and real estate. However, these analytics have recently begun to be made available by companies such as QLS Technologies (https://qlstechnologies.com), which should facilitate the process by which the risk and reward of true megafund investments can be more accurately assessed.

## 5. Conclusion

In this article, we introduce the concept of a global megafund designed to transform the financing of drug development by pooling investments across multiple stages of clinical progress and therapeutic areas worldwide. Using Monte Carlo simulation, we demonstrate that even a simple equal-weighted strategy delivers strong financial performance, achieving an annualized ROE of 12.0% with a Sharpe ratio of 1.37. For equity investors, this return is comparable to the long-term historical performance of the S&P 500, while for fixed-income investors, the senior and junior tranches exhibit default probabilities of only 0.030% and 0.116%, comparable to AAA- and AA-rated bonds. Taken together, these results establish the megafund as a powerful and reliable vehicle for investors seeking opportunities beyond traditional financial assets.

Beyond strictly financial metrics, however, the HNF also displays a potentially transformational effect on global healthcare. On average, the megafund supports the development of 25 novel therapies, potentially reaching 44 million patients worldwide and addressing an estimated 244 million DALYs. By mobilizing large-scale capital into critical areas of unmet medical need, the megafund offers not only sustainable returns but also a profound contribution to global public health. This dual capacity—delivering competitive financial performance while catalyzing the creation of life-saving treatments—underscores the promise of the megafund model as a next-generation framework for aligning capital markets with social impact. Evidence from existing vehicles, such as the GHIF, suggests that this dual mandate is achievable in practice. GHIF’s $108 million portfolio of late-stage products demonstrates that blended public, philanthropic, and private capital can be mobilized to deliver both financial returns and substantial health impact [17]. The design of the HNF, beyond its equal-weighted benchmark, also incorporates a quadratic optimization framework that explicitly integrates the impact of healthcare into its asset allocation strategy. This optimal portfolio construction framework demonstrates that structured, multi-objective optimization can further amplify both the financial performance and the social impact of a diversified megafund. In particular, by appropriately assigning portfolio weights, the fund can be systematically adjusted to prioritize higher returns, lower risk, or greater healthcare impact according to the preference of investors.

Through a series of sensitivity analyses, we demonstrate the robustness of megafund performance across a wide range of assumptions. Varying key parameters such as the leverage ratio, the PoS, the market value of approved drugs, and the cost of capital consistently confirms the resilience of the HNF to structural changes in the drug development environment. While a higher leverage ratio amplifies both the megafund’s returns and risks, a lower leverage ratio provides the megafund with greater financial stability and ensures more drug approvals. Similarly, improvements in clinical success rates or drug market values lead to stronger financial performance and enhanced healthcare outcomes. Even under conservative assumptions, the fund can sustain double-digit annualized equity returns with a low probability of default, underscoring its strength as a reliable investment vehicle and a powerful driver of global healthcare impact.

In conclusion, the proposed HNF underscores the potential for the megafund model to finance global needs in biomedical innovation that are currently unmet by present-day financing sources and structures. The HNF is resilient to structural changes in the drug development landscape. Its baseline performance is strong, and its healthcare impact due to new therapeutics represents the equivalent of hundreds of millions of additional years of human life. As a global investment vehicle, the Health of Nations Fund is poised to make substantial improvements to healthcare outcomes worldwide.

## Supporting information

S1 Text Megafund simulation parameters. The construction of the HNF relies on a wide range of parameters. This appendix describes these parameters and provides the values used in our simulation.
(PDF)
