# Supplementary material for: The Health of Nations Fund: Financing global drug development
Source: PLOS Glob Public Health. 2026 Jun 16;6(6):e0005248. doi: 10.1371/journal.pgph.0005248 (PMC13271462; doi:10.1371/journal.pgph.0005248)
Supplement: S1 Text — The construction of the HNF relies on a wide range of parameters. This appendix describes these parameters and provides the values used in our simulation. (PDF) [file pgph.0005248.s001.pdf]

S1 Text: Megafund Simulation Parameters

The construction of the HNF relies on a wide range of parameters. In this appendix, we describe these parameters and provide the values used in our simulation.

A Probabilities of success

We use the compiled probabilities of successful phase transitions from phase 1 to phase 2, phase 2 to phase 3, and phase 3 to NDA as estimated by Project ALPHA in the second quarter of 2024.[1] The probability of transition from the preclinical phase to phase 1 is taken from Paul et al. [1], while the probability from NDA status to regulatory approval is set as the average of the estimates reported by Hay et al. [2] and Thomas et al. [3]. Table A summarizes the probabilities of success used in our simulation.

Table A: **Estimated probabilities of successful phase transitions (%).**

Therapeutic Area	Preclinical to Phase 1	Phase 1 to Phase 2	Phase 2 to Phase 3	Phase 3 to NDA	NDA to Approval
Oncology	69.0	63.2	28.6	57.7	82.1
Metabolic/Endocrinology	69.0	67.2	44.9	67.5	84.4
Cardiovascular	69.0	73.6	54.2	60.5	84.4
Central Nervous System	69.0	67.7	43.9	60.6	83.9
Autoimmune/Inflammation	69.0	67.8	50.9	72.3	83.2
Genitourinary	69.0	75.3	54.7	69.9	83.1
Infectious Disease	69.0	72.2	61.5	76.5	86.8
Ophthalmology	69.0	89.1	54.3	46.9	79.0

B Costs of clinical trials

The cost estimates for phase 1, phase 2, and phase 3 clinical trials are taken from Sertkaya et al. [4], while the cost of preclinical R&D is obtained from Strovel et al. [5]. The cost of submitting an NDA is based on the Prescription Drug User Fee Act (PDUFA). All estimates are then adjusted to 2025 U.S. dollars using the Biomedical Research and Development Price Index (BRDPI). The final costs applied in our simulation are reported in Table B.

C Durations of clinical trials

The duration estimates for the typical phase 1, phase 2, and phase 3 clinical trial are taken from Wong, Siah, and Lo [6, 7]. The duration of preclinical R&D is obtained from Fernandez, Stein, and Lo [8], while the NDA review period is based on the timeline set by the PDUFA. Table C summarizes the durations used in our simulation.

[1] See `https://projectalpha.mit.edu/pos-archive/2024q2/`.

Table B: **Estimated costs of clinical trials ($ million).**

Therapeutic Area	Preclinical	Phase 1	Phase 2	Phase 3	NDA
Oncology	3.9	11.2	27.8	27.4	2.9
Metabolic/Endocrinology	3.9	3.5	30.0	21.1	2.9
Cardiovascular	3.9	5.5	17.4	31.2	2.9
Central Nervous System	3.9	9.7	34.5	23.8	2.9
Autoimmune/Inflammation	3.9	9.9	40.9	40.2	2.9
Genitourinary	3.9	7.7	36.2	21.7	2.9
Infectious Disease	3.9	10.4	35.2	28.3	2.9
Ophthalmology	3.9	13.1	34.2	38.0	2.9

Table C: **Estimated durations of clinical trials (months).**

Therapeutic Area	Preclinical	Phase 1	Phase 2	Phase 3	NDA
Oncology	12.0	36.4	41.0	49.9	10.0
Metabolic/Endocrinology	12.0	10.8	31.5	32.5	10.0
Cardiovascular	12.0	12.6	34.2	40.3	10.0
Central Nervous System	12.0	11.1	31.1	34.5	10.0
Autoimmune/Inflammation	12.0	11.2	32.7	32.6	10.0
Genitourinary	12.0	12.6	26.2	33.5	10.0
Infectious Disease	12.0	18.7	31.7	35.6	10.0
Ophthalmology	12.0	18.2	27.4	34.3	10.0

D Market values of approved drugs

The estimates of market value of an approved drug are calculated as the present value of a perpetual annuity, where the annual global market size of each drug is obtained from various market research reports and discounted at a rate of 14%. Table D summarizes the market values of drugs across different therapeutic areas used in our simulation.

Table D: **Estimated market values of drugs ($ million).**

Therapeutic Area	Market Value
Oncology	1,063.0
Metabolic/Endocrinology	1,386.1
Cardiovascular	1,233.0
Central Nervous System	1,056.8
Autoimmune/Inflammation	1,883.5
Genitourinary	437.5
Infectious Disease	699.0
Ophthalmology	855.6

E Costs of capital for project valuation

The estimated costs of capital are taken from Avance and adjusted to the 2024 discount rate by adding 5.25%, the change in the U.S. policy rate between May 2021 and August 2024. Table E summarizes the costs of capital for project valuation used in our simulation.

Table E: **Estimated costs of capitals for project valuation (%).**

	Preclinical	Phase 1	Phase 2	Phase 3	NDA
Cost of Capital	23.9	23.9	21.3	18.9	18.9

F Costs of project acquisition

The fair value of projects in an intermediate stage x, denoted V_x, is obtained using Equation (6). We then estimate the acquisition cost of a project as the sum of V_x and the present value of all future clinical trial costs, which serves as a reserve for future expenses:

$$\text{Expected acquisition cost} = V_x + \sum_{s=x}^{\text{NDA}} C_s p_{x:s},$$

where s represents the different stages (preclinical, phase 1, phase 2, phase 3, and NDA), and $p_{x:s}$ is the probability of successfully transitioning from stage x to stage s. Following [7], $p_{x:s}$ is estimated as the product of all phase transition probabilities between x and s given in Section A. Table F reports the expected acquisition costs of projects across therapeutic areas used in our simulation.

Table F: **Expected costs of acquiring projects ($ million).**

Therapeutic Area	Preclinical	Phase 1	Phase 2	Phase 3	NDA
Oncology	27.3	34.0	36.1	213.4	755.3
Metabolic/Endocrinology	43.8	71.3	120.2	428.9	1,013.1
Cardiovascular	40.9	66.0	103.4	306.0	900.7
Central Nervous System	33.3	50.0	80.1	284.0	767.6
Autoimmune/Inflammation	71.2	115.3	193.7	613.9	1,356.2
Genitourinary	34.7	44.7	53.5	136.6	314.7
Infectious Disease	40.6	61.1	96.9	241.8	525.4
Ophthalmology	47.1	62.7	66.4	168.1	584.9

G Correlation between projects

Drugs within the same therapeutic area tend to share similar characteristics, leading to correlated outcomes in clinical trials, while projects in different therapeutic areas are expected

to exhibit weak correlations. To account for these interdependencies, we assume a correlation of 0.05 across projects from different therapeutic areas, and a higher correlation of 0.20 across projects within the same therapeutic area. This approach allows us to realistically model the dependence structure between clinical trial outcomes.

H Cash flows of royalty assets

Table H summarizes the projected cash flows of the royalty assets incorporated into the megafund. We assume that the royalty assets generate cash flows for ten years, with sales expanding rapidly in the first five years before stabilizing to a more moderate growth trajectory thereafter. The royalty rate is set at 5% of total sales. Discounting the expected royalty payments at a rate of 13.75% yields a present value of $200 million.

Table H: **Cash flows of the royalty assets ($ million).**

Year	1	2	3	4	5	6	7	8	9	10
Sales	200	402	604	805	1,007	1,058	1,111	1,166	1,224	1,286
Royalty (5% of Sales)	10.0	20.1	30.2	40.3	50.4	52.9	55.6	58.3	61.2	64.3

I DALYs

We use the average DALYs for each therapeutic area, obtained from the 2021 Global Burden of Disease study [9]. Table I summarizes the average DALYs across the different therapeutic areas used in our simulation.

Table I: **Average DALYs of different therapeutic areas (per 1 million population).**

Therapeutic Area	DALY
Oncology	14.8
Metabolic/Endocrinology	7.9
Cardiovascular	22.6
Central Nervous System	12.8
Autoimmune/Inflammation	10.2
Genitourinary	4.5
Infectious Disease	11.0
Ophthalmology	3.0

References

[1] Steven M Paul, Daniel S Mytelka, Christopher T Dunwiddie, Charles C Persinger, Bernard H Munos, Stacy R Lindborg, and Aaron L Schacht. How to improve R&D

productivity: the pharmaceutical industry's grand challenge. *Nature Reviews Drug Discovery*, 9(3):203–214, 2010.

[2] Michael Hay, David W Thomas, John L Craighead, Celia Economides, and Jesse Rosenthal. Clinical development success rates for investigational drugs. *Nature Biotechnology*, 32(1):40–51, 2014.

[3] David W Thomas, Justin Burns, John Audette, Adam Carroll, Corey Dow-Hygelund, and Michael Hay. Clinical development success rates 2006–2015. BIO Industry Analysis, 2016.

[4] Aylin Sertkaya, Hui-Hsing Wong, Amber Jessup, and Trinidad Beleche. Key cost drivers of pharmaceutical clinical trials in the United States. *Clinical Trials*, 13(2):117–126, 2016.

[5] Jeffrey Strovel, Sitta Sittampalam, Nathan P Coussens, Michael Hughes, James Inglese, Andrew Kurtz, Ali Andalibi, Lavonne Patton, Chris Austin, Michael Baltezor, Michael Beckloff, Michael Weingarten, and Scott Weir. Early drug discovery and development guidelines: for academic researchers, collaborators, and start-up companies. Assay Guidance Manual [Internet], 2016.

[6] Chi Heem Wong, Kien Wei Siah, and Andrew W Lo. Estimating clinical trial success rates and related parameters in oncology. Available at SSRN 3355022, 2019.

[7] Chi Heem Wong, Kien Wei Siah, and Andrew W Lo. Estimation of clinical trial success rates and related parameters. *Biostatistics*, 20(2):273–286, 2019.

[8] Jose-Maria Fernandez, Roger M Stein, and Andrew W Lo. Commercializing biomedical research through securitization techniques. *Nature Biotechnology*, 30(10):964–975, 2012.

[9] Global Burden of Disease (GBD). Cause of death or injury. `https://vizhub.healthdata.org/gbd-results/`, 2021.
